# Clinical impact of advanced chronic kidney disease in patients with non-HIV pulmonary cryptococcosis

**DOI:** 10.1186/s12890-020-1149-3

**Published:** 2020-04-29

**Authors:** Hiroki Tashiro, Tetsuro Haraguchi, Koichiro Takahashi, Hironori Sadamatsu, Ryo Tajiri, Ayako Takamori, Shinya Kimura, Naoko Sueoka-Aragane

**Affiliations:** 1 Division of Hematology, Respiratory Medicine and Oncology, Department of Internal Medicine, Faculty of Medicine, Saga University, 5-1-1 Nabeshima, Saga, Saga Prefecture 849-8501 Japan; 2 Clinical Research Center, Saga University Hospital, Saga, Japan

**Keywords:** Pulmonary cryptococcosis, Chronic kidney disease, Clinical manifestations, Dissemination

## Abstract

**Background:**

Pulmonary cryptococcosis is an uncommon infectious disease that can develop in both immunocompromised and immunocompetent patients. The severity of chronic kidney disease (CKD) was reported to be one of the risk factors for pulmonary cryptococcosis, but its clinical characteristics have not been fully assessed. The purpose of this study was to clarify the clinical characteristics of advanced CKD in patients with pulmonary cryptococcosis.

**Methods:**

The present study retrospectively investigated 56 patients who had non-human immunodeficiency virus (HIV) pulmonary cryptococcosis and were treated at Saga University Hospital between 2005 and 2018. The clinical characteristics were evaluated and compared between patients with estimated glomerular filtration rate (eGFR) > 45 mL/min/1.73 m^2^ (*n* = 42, early CKD) and those with eGFR < 45 mL/min/1.73 m^2^ (*n* = 14, advanced CKD.

**Results:**

Compared with patients with early CKD, those with advanced CKD had significantly higher rate of disseminated cryptococcosis (21.4% vs. 2.4%, *p* = 0.03); lower percentage of patients who recovered after treatment (63.6% vs. 92.5%, *p* = 0.02); and more frequent clinical features of fever (57.1% vs. 19.0%, *p* < 0.01), pleural effusion (21.4% vs. 2.4%, *p* = 0.03), high white blood cell count (8550/mL vs. 6150/mL, *p* = 0.01) and C-reactive protein (CRP) (2.1 mg/dL vs. 0.2 mg/dL, *p* = 0.02), and low level of serum albumin (3.0 g/dL vs. 3.8 g/dL, *p* < 0.01). Multivariate analysis adjusted by immunosuppressive drug use indicated the significant factors of fever (odds ratio or β value [95% confidence interval] 6.4 [1.65–20.09], *p* < 0.01), high white blood cell count (1293.2 [110.2–2476.2], *p* = 0.03), C-reactive protein (0.89 [0.18–1.59], *p* = 0.01) and low level of serum albumin (− 0.34 [− 0.54 – − 0.14], *p* < 0.01) in patients with eGFR < 45 mL/min/1.73m^2^.

**Conclusion:**

Advanced CKD was associated with poor clinical characteristics and outcomes in patients with non-HIV pulmonary cryptococcosis.

**Trial registration:**

The patients in this study were registered retrospectively.

## Background

Cryptococcosis is an uncommon infectious disease caused by *Cryptococcus neoformans* and *Cryptococcus gattii* [1, 2]. The fungus mainly infects the lungs and central nervous system, but it can less frequently affect other organs, including the eyes, prostate, skin, and bone, in both immunocompetent and immunocompromised patients [3–5]. Occasionally, cryptococcus can disseminate to several organs and lead to mortality, depending on the host immunity [6–8].

Pulmonary cryptococcosis is important, because the respiratory tract is the most common portal of entry [9, 10]. Several studies have reported that the clinical manifestations, including symptoms, laboratory data, radiologic findings, dissemination, and outcome, of pulmonary cryptococcosis were different in patients with immunocompromised comorbidities, such as human immunodeficiency virus (HIV) infection, diabetes mellitus, malignancy, organ transplantation, immunosuppressive treatment use, and chronic kidney disease (CKD) [5, 11–14]. CKD is one of the essential health conditions and is defined by an estimated glomerular filtration rate (eGFR) of < 60 mL/min/1.73 m^2^ with positive findings of urinary protein that persists for 3 months [15, 16]. A large cohort study identified that an eGFR of < 45 mL/min/1.73 m^2^ was related with all-cause and cardiovascular mortality [17]. There is increasing evidence that CKD is closely linked with host immune systems [18] and the risk for infections [19]. Some reports identified that the severity of CKD was involved with the clinical characteristics of pulmonary cryptococcosis [20, 21]; however, the clinical features have not been fully assessed.

The purpose of this study was to clarify the clinical characteristics of advanced CKD in patients with pulmonary cryptococcosis. We investigated 56 patients with non-HIV pulmonary cryptococcosis and compared the clinical manifestations between 42 patients with eGFR > 45 mL/min/1.73 m^2^ and 14 patients with eGFR < 45 mL/min/1.73 m^2^. We identified that the symptoms, laboratory findings, radiologic patterns, and outcome, including dissemination, were different between the 2 groups and were worse in patients with pulmonary cryptococcosis and advanced CKD. These results may practically contribute to the clinical risk evaluation for pulmonary cryptococcosis.

## Methods

### Patients and setting

We retrospectively evaluated 56 patients diagnosed as pulmonary cryptococcosis without HIV infection at Saga University Hospital between 2005 and 2018. This study was approved by the ethics committee of Saga University Hospital (approval number: 2019-09-06, approval date: Nov 25, 2019) and was performed in accordance with the 1964 Declaration of Helsinki.

The assessment of pulmonary cryptococcosis was made by 1) clinical diagnosis, based on serum anticryptococcal antigen and chest radiologic abnormalities; 2) histologic diagnosis, based on the presence of cryptococcal pathogen on Grocott’s staining of lung histologic samples and chest radiologic abnormalities; and 3) pathogenic diagnosis, based on the detection of cryptococcus species on airway, blood, or cerebrospinal fluid culture and chest radiologic abnormalities. All of the species detected from the cultured samples were *C. neoformans*. Disseminated cryptococcosis was defined as isolation of *C. neoformans* from blood, sterile body fluid, or any extrapulmonary site [8]. Upon the diagnosis of pulmonary cryptococcosis, the clinical manifestations, including comorbidities, symptoms, laboratory data, and radiologic findings, were collected from the medical records. The eGFR was calculated by the following formula: 194 × serum creatinine^-1.094^ × age^-0.287^ × 0.739 (for females) [22]. In all patients with eGFR < 45 mL/min/1.73 m^2^, the persistence of the same renal function for more than 3 months was verified, according to the CKD guidelines [15, 17].

To address the clinical impact of severe CKD on pulmonary cryptococcosis, we divided 56 patients with pulmonary cryptococcosis into those with eGFR > 45 mL/min/1.73 m^2^ (early CKD) and those with eGFR < 45 mL/min/1.73 m^2^ (advanced CKD). An eGFR of 45 mL/min/1.73 m^2^ was set as the cutoff, based on a previous report that identified its relationship with mortality [17]. Treatment, including the intake of antifungal drugs or surgery, was based on the physician’s judgment. The outcome of nonrecovery from pulmonary cryptococcosis after 3 months of antifungal drug treatment was defined as death or worsening radiologic and clinical findings.

### Comorbidities

The predisposing factors, including pulmonary diseases, diabetes mellitus, connective tissue diseases, malignancy, immunosuppressive drug use, and hemodialysis, were collected from the medical records. There were no patients who received organ transplantation.

### Radiologic findings

In the present study, high-resolution computed tomography (HRCT) of the chest upon the diagnosis of pulmonary cryptococcosis was evaluated by 2 pulmonologists, who referred to the official reports from a radiologist. The most dominant chest radiologic finding was classified based on the following patterns: [1] single nodule (< 30 mm), [2] multiple nodules, [3] mass (> 30 mm), [4] cavitation, [5] consolidation, [6] ground glass attenuation, or [7] pleural effusion.

### Statistical analysis

The clinical data were analyzed by Wilcoxon rank sum test for continuous variables or chi-square test for categorical variables. Laboratory parameters, such as CRP, were analyzed after log transformation, considering a skewed to the right distribution. The present study found that immunosuppressive drug was used more often by patients with advanced CKD than by those with early CKD. Because this might affect the present results, we performed multivariate analysis for the variables, including fever, white blood cell count, serum albumin, and CRP, which were significantly different between patients with preserved eGFR and those with declined eGFR in the univariate analysis (data not shown in the tables). Disseminated cryptococcosis, pleural effusion, and recovery after treatment were not assessed because of the small sample size. The comparative results were adjusted by the predisposing variable of immunosuppressive drug use. For multivariate analyses, logistic regression was performed for categorical variables and multiple linear regression was performed for continuous variables. Quantitative data were presented as mean or ± standard deviation (SD) or median ± interquartile range (IQR); odds ratio (OR) or coefficient β value was calculated. The two-tailed significance level was set at *p* < 0.05. Statistical analysis was performed with JMP Pro version 14.2.0 software (SAS Institute Inc., Cary, NC, USA).

## Results

### Clinical characteristics and comorbidities in patients with pulmonary cryptococcosis

There were 42 patients with early CKD and 14 patients with advanced CKD. Compared with the group with early CKD, the group with advanced CKD had similar age, sex, and body mass index; tended to have higher number of patients with pathogenic diagnosis; had significantly higher rate of disseminated pulmonary cryptococcosis; similar comorbidities of pulmonary diseases, diabetes mellitus, connective tissue diseases, and malignancy; and more frequent use of immunosuppressive drugs. In the present study, only 1 patient in the early CKD group was on hemodialysis (Table 1).

**Table 1:** Epidemiologic and clinical characteristics of patients pulmonary nocardiosis, according to eGFR (*n* = 56)

	eGFR > 45	eGFR ≤45	*p* value
n	42	14	
Age (years) ^a^	67.4 ± 2.5	67.7 ± 2.9	0.55
Gender (Male/Female)	21/21	6/8	0.64
BMI (kg/m^2^) ^a^	22.2 ± 0.7	22.2 ± 1.7	0.53
Diagnosis			
Clinical	24 (57.1%)	7 (50.0%)	0.64
Histological	10 (23.8%)	2 (14.3%)	0.44
Pathogenic	10 (23.8%)	7 (50.0%)	0.07
Disseminated cryptococcosis	1 (2.4%)	3 (21.4%)	0.03
Comorbidity			
Pulmonary diseases	4 (9.5%)	1 (7.1%)	0.78
Diabetes mellitus	12 (28.6%)	6 (42.9%)	0.33
Connective tissue diseases	4 (9.5%)	2 (14.3%)	0.63
Malignancy	12 (28.6%)	2 (14.3%)	0.26
Immunosuppressive drug use	14 (33.3%)	9 (64.3%)	0.04
On hemodialysis	0 (0.0%)	1 (7.1%)	0.09

*eGFR* estimated glomerular filtration rate, *BMI* body mass index

^a^ Data are presented as mean ± standard deviation

### Comparison of symptoms and laboratory findings in patients with pulmonary cryptococcosis

Compared with the group with early CKD, the group with advanced CKD had similar percentage of asymptomatic patients; similar pulmonary cryptococcosis-related symptoms of cough, sputum, chest pain, and dyspnea; significantly higher percentage of patients who had fever (> 37.5 °C) (57.1% vs. 19.0%, *p* < 0.01); and, on laboratory data, significantly higher white blood cell count (8550/mL vs. 6150/mL, *p* = 0.01) and CRP level (2.1 mg/dL vs. 0.2 mg/dL, *p* = 0.02) but significantly lower serum albumin (3.0 g/dL vs. 3.8 g/dL, *p* < 0.01) and similar lymphocyte count, serum calcium, immunoglobulin G, and anticryptococcal antigen (Table 2).

**Table 2:** Comparison of the symptoms and laboratory data in patients with pulmonary cryptococcosis, according to eGFR

	eGFR > 45	eGFR ≤45	*p* value
n	42	14	
Asymptomatic	15 (35.7%)	2 (14.3%)	0.11
Cough	12 (28.6%)	6 (42.9%)	0.32
Sputum	6 (14.3%)	4 (28.6%)	0.24
Chest pain	4 (9.5%)	1 (7.1%)	0.78
Fever (> 37.5 °C)	8 (19.0%)	8 (57.1%)	< 0.01
Dyspnea	2 (4.8%)	1 (7.1%)	0.74
Laboratory data			
WBC (/mL)^a^	6150 ± 5100	8550 ± 7575	0.01
Lymphocyte cell (/mL)^a^	1151.6 ± 740.4	1326.9 ± 518.0	0.87
Serum albumin (g/dL)^a^	3.8 ± 3.4	3.0 ± 2.6	< 0.01
Serum calcium^a b^	9.6 ± 9.0	9.3 ± 9.1	0.88
Immunoglobulin G (mg/dL)^a^	903.5 ± 745.3	1233.5 ± 621.5	0.48
CRP (mg/dL)^a^	0.2 ± 0.1	2.1 ± 0.2	0.02
Anti-cryptococcus antigen^a^	16 ± 1	8 ± 5	0.48

*WBC* White blood cell, *CRP* C reactive protein, *eGFR* estimated glomerular filtration rate

^a^ Data are presented as median ± interquartile range

^b^ corrected by serum albumin

### Comparison of the radiologic findings on HRCT in patients with pulmonary cryptococcosis

According to previous reports on the different distribution and patterns of radiologic findings in pulmonary cryptococcosis, depending on the immune status [12, 23], we evaluated and compared the area and features of pulmonary abnormalities between patients with early CKD and those with advanced CKD. Compared with the group with early CKD, the group with advanced CKD had lower number of patients whose pulmonary abnormalities were limited to 1 lobe (28.6% vs. 57.1%, *p* = 0.06) and were distributed in only a unilateral lung field (50.0% vs. 76.2%, *p* = 0.07); similar patterns of single nodule, multiple nodules, masses, cavitation, consolidation, and ground glass attenuation; and significantly higher number of patients with pleural effusion (21.4% vs. 2.4%, *p* = 0.03) (Table 3).

**Table 3:** Comparison of the radiologic features on high-resolution computed tomography in patients with pulmonary cryptococcosis, according to eGFR

	eGFR > 45	eGFR ≤45	*p* value
Limited in one lobe	24 (57.1%)	4 (28.6%)	0.06
Unilateral lung field	32 (76.2%)	7 (50.0%)	0.07
Single nodule	6 (14.3%)	1 (7.1%)	0.46
Multiple nodules	27 (64.3%)	10 (71.4%)	0.62
Mass	3 (7.1%)	1 (7.1%)	1
Cavitation	6 (14.3%)	1 (7.1%)	0.46
Consolidation	8 (19.0%)	3 (21.4%)	0.85
Ground glass attenuation	0 (0.0%)	1 (7.1%)	0.09
Pleural effusion	1 (2.4%)	3 (21.4%)	0.03

*eGFR* estimated glomerular filtration rate

### Treatment and outcome of patients with pulmonary cryptococcosis

The number of patients who took antifungal drugs and the duration of antifungal drug treatment were not different between the 2 groups. Azole was the antifungal drug used by 94.1% of patients with early CKD and 75.0% of patients with advanced CKD. In 25% of patients with advanced CKD, amphotericin B was administered for dissemination or coinfection with aspergillus. Surgery tended to be performed more frequently for patients with early CKD than for those with advanced CKD (12.8% vs. 0%, *p* = 0.09). Evaluation of the clinical outcomes of 40 patients with early CKD and 11 patients with advanced CKD showed that the rate of recovery after treatment was significantly higher in the former than in the latter (92.5% vs. 63.6%, *p* = 0.02) (Table 4). Among early CKD patients who did not recover, 2 died and 1 developed exacerbation secondary to fluconazole but recovered after changing the treatment to voriconazole. All 4 patients with advanced CKD died.

**Table 4:** Comparison of the treatment and outcome of patients with pulmonary cryptococcosis, according to eGFR

	eGFR > 45	eGFR ≤45	*p* value
No treatment	3 (7.1%)	2 (14.3%)	0.44
Treatment			
Antifungal drugs	34/39 (87.2%)	12/12 (100%)	0.09
Treatment duration (months) ^a^	8.9 ± 1.7	11.0 ± 2.8	0.53
Surgery	5/39 (12.8%)	0/12 (0.0%)	0.09
Outcome			
Recovered*	37/40 (92.5%)	7/11 (63.6%)	0.02

*eGFR* estimated glomerular filtration rate

*2 patients with eGFR > 45 and 3 patients with eGFR ≤45 were not evaluated because of no information about the outcome

^a^ Data are presented as mean ± standard deviation

### Multivariate analysis of the clinical impact of CKD on pulmonary cryptococcosis

Previous studies reported that immunocompromising comorbidities, such as diabetes mellitus, malignancy, and immunosuppressive drug use, were associated with clinical characteristics [5, 11, 12]. The present study found that immunosuppressive drug was used more often by patients with advanced CKD than by those with early CKD (Table 1). Because this might have affected the results, we performed multivariate analysis for the variables that were significantly different between patients with early CKD and those with advanced CKD. In the models that were stratified according to eGFR and use of immunosuppressive drug, the variables included fever, white blood cell count, serum albumin, and CRP. Disseminated cryptococcosis, pleural effusion, and recovery after treatment were not assessed because of the small sample size. On multivariate analysis, fever, white blood cell count, serum albumin, and CRP remained significantly different between patients with early CKD and those with advanced CKD, even after adjustment by the use of immunosuppressive drug (Table 5).

**Table 5:** Multivariate analysis adjusted according to eGFR ≤45 mL/min/1.73 m^2^ and use of immune suppressive drug

	Multivariate analysis					
	eGFR ≤45			Immunosuppressive drug use		
	ORorβ	95%CI	*p* value	ORorβ	95%CI	*p* value
Fever (> 37.5 °C)	6.4	1.65–20.09	< 0.01	0.7	0.19–2.56	0.59
Laboratory data						
WBC (/mL)	1293.2	110.2–2476.2	0.03	598.9	− 442.4 – 1640.1	0.25
Serum albumin (g/dL)	−0.34	−0.54 – −0.14	< 0.01	−0.06	−0.22 – −0.11	0.52
CRP (mg/dL)	0.89	0.18–1.59	0.01	0.12	−0.5 – 0.74	0.7

Fever, white blood cell count, albumin and CRP which were significantly different between patients with eGFR> 45 and those with eGFR ≤45, and the comparative results were adjusted by predisposing variable of immunosuppressive drug use

*eGFR* estimated glomerular filtration rate, *WBC* White blood cell, *CRP* C reactive protein, *OR* odds ratio, *β* standardized β value, *CI* Confidence interval

## Discussion

In the present study, we analyzed 56 patients with non-HIV pulmonary cryptococcosis and evaluated the clinical characteristics depending on the CKD severity. We identified that the rates of disseminated cryptococcosis and nonrecovery after treatment were significantly higher in patients with advanced CKD than in those with early CKD. For the clinical features, fever, pleural effusion, high white blood cell count and CRP, and low level of serum albumin were more frequently seen in patients with advanced CKD than in those with early CKD. To our best knowledge, this was the first report to clarify the clinical manifestations of advanced CKD in patients with non-HIV pulmonary cryptococcosis.

CKD is one of the risk factors for pulmonary cryptococcosis; this statement can be supported by several individual cases that had severe clinical manifestations, such as dissemination and mortality, in chronic renal failure, especially when under dialysis [14, 20]. Pyrgos V et al. reported that compared with all hospitalized patients, those hospitalized for cryptococcus meningitis had significantly more frequent comorbidities of acute (28.5%) and chronic renal failure (14.3%) [24]. Moreover, Hung MS et al. identified that fever and pleural effusion were significantly more frequent in disseminated pulmonary cryptococcosis than in localized pulmonary cryptococcosis [8]. Similarly, our findings showed higher rates of disseminated cryptococcosis and nonrecovery after treatment in advanced CKD patients with pulmonary cryptococcosis complicated by fever and pleural effusion.

The mechanisms of the association between CKD severity and the risk for pulmonary cryptococcosis remain unclear. However, some possible mechanisms, particularly the effect of the T cell response on the risk for pulmonary cryptococcosis and the pathophysiology of CKD, had been reported. HIV, which is characterized by a decline in CD4^+^ T cells, is one of the major causes of cryptococcosis [25]. Moreover, the clinical characteristics and outcome of pulmonary cryptococcosis in patients with immunocompromising conditions, such as underlying malignancy, immunosuppressive drug use, and diabetes mellitus, were reported to be different from those in immunocompetent patients [12, 13, 23]. Likewise, CKD has been associated with inactivation of the T cell response and induction of T cell apoptosis [26, 27], which cause immune dysfunction and increase the risk for infection [18, 19]. Notably, host response to cryptococcal infection involves helper T cell response with the production of cytokines, including tumor necrosis factor, interferon-ɤ, and interleukin 2 [28, 29]. According to these data, we considered that the immune dysfunction in advanced CKD might have affected the clinical characteristics and outcomes of patients with pulmonary cryptococcosis.

The clinical characteristics, including symptoms, laboratory abnormalities, and radiologic features, in pulmonary cryptococcosis are variable [30]. Consistent with our results, the absence of symptoms was reported to be relatively frequent in immunocompetent patients, whereas the pulmonary cryptococcosis-associated symptoms, especially fever and chest pain, were markedly seen in immunocompromised hosts [12, 31]. The radiologic patterns and distribution in pulmonary cryptococcosis have been closely related with host immunity. Nodules have been more frequently observed in immunocompetent patients, whereas pleural effusion, cavitation, and consolidation in a large lung area have been markedly observed in immunocompromised patients [31–34]. Our results of more frequent pulmonary CT abnormalities that were limited to 1 lobe and in a unilateral lung field in patients with early CKD than in those with advanced CKD supported these previous data and the involvement of immunodeficiency secondary to CKD. In addition, we clarified that high white blood cell count and CRP and low serum albumin might be biomarkers for risk evaluation in patients with pulmonary cryptococcosis and advanced CKD.

There were 2 limitations in the present study. First, treatment, including the administration of antifungal drugs and surgery, was upon the discretion of the physician, and this might have affected the clinical outcomes. Second, the present study was performed on a small number of patients at a single hospital with limited ethnic diversity. To confirm the validity of our results, multicenter prospective studies with larger number of patients should be performed.

## Conclusions

Compared with early CKD, advanced CKD was associated with significantly higher rates of disseminated cryptococcosis and nonrecovery after treatment and more frequent clinical features of fever, pleural effusion, high white blood cell count and CRP, and low level of serum albumin. These results may practically contribute to the clinical risk evaluation of patients with pulmonary cryptococcosis.

## Data Availability

The datasets used and analyzed during the present study are available from the corresponding author on reasonable request.

## Abbreviations

CKD: Chronic kidney disease
HIV: Human immunodeficiency virus
eGFR: Estimated glomerular filtration rate
HRCT: High-resolution computed tomography
CRP: C-reactive protein
SD: Standard deviation
IQR: Interquartile range
WBC: White blood cell
OR: Odds ratio
β: Standardized β value
CI: Confidence interval
